# Health Care Workers’ Motivations for Enrolling in Massive Open Online Courses During a Public Health Emergency: Descriptive Analysis

**DOI:** 10.2196/51915

**Published:** 2024-06-19

**Authors:** Jennifer Jones, Jamie Sewan Johnston, Ngouille Yabsa Ndiaye, Anna Tokar, Saumya Singla, Nadine Ann Skinner, Matthew Strehlow, Heini Utunen

**Affiliations:** 1 Stanford School of Medicine, Stanford, CA, United States; 2 Stanford Center for Health Education, Stanford, CA, United States; 3 Learning and Capacity Development Unit, Health Emergencies Programme, World Health Organization, Geneva, Switzerland

**Keywords:** massive open online course, MOOC, online learning, online courses, online course, health care education, medical education, education, training, professional development, continuing education, COVID-19 training, infectious disease outbreak response, emergency, public health, crisis, crises, outbreak, pandemic, COVID-19, SARS-CoV-2, coronavirus, humanitarian emergency response, health care workers, nurse, nurses, practitioner, practitioners, clinician, clinicians, health care worker, medic, low-income, lower-middle income, LIC, LMIC, developing country, developing countries, developing nation, developing nations, case study, survey, surveys, descriptive analysis, descriptive analyses, motivation, motivations, lower-middle–income country, low-income country

## Abstract

**Background:**

Massive open online courses (MOOCs) are increasingly used to educate health care workers during public health emergencies. In early 2020, the World Health Organization (WHO) developed a series of MOOCs for COVID-19, introducing the disease and strategies to control its outbreak, with 6 courses specifically targeting health care workers as learners. In 2020, Stanford University also launched a MOOC designed to deliver accurate and timely education on COVID-19, equipping health care workers across the globe to provide health care safely and effectively to patients with the novel infectious disease. Although the use of MOOCs for just-in-time training has expanded during the pandemic, evidence is limited regarding the factors motivating health care workers to enroll in and complete courses, particularly in low-income countries (LICs) and lower-middle–income countries (LMICs).

**Objective:**

This study seeks to gain insights on the characteristics and motivations of learners turning to MOOCs for just-in-time training, to provide evidence that can better inform MOOC design to meet the needs of health care workers. We examine data from learners in 1 Stanford University and 6 WHO COVID-19 courses to identify (1) the characteristics of health care workers completing the courses and (2) the factors motivating them to enroll.

**Methods:**

We analyze (1) course registration data of the 49,098 health care workers who completed the 7 focal courses and (2) survey responses from 6272 course completers. The survey asked respondents to rank their motivations for enrollment and share feedback about their learning experience. We use descriptive statistics to compare responses by health care profession and by World Bank country income classification.

**Results:**

Health care workers completed the focal courses from all regions of the world, with nearly one-third (14,159/49,098, 28.84%) practicing in LICs and LMICs. Survey data revealed a diverse range of professional roles among the learners, including physicians (2171/6272, 34.61%); nurses (1599/6272, 25.49%); and other health care professionals such as allied health professionals, community health workers, paramedics, and pharmacists (2502/6272, 39.89%). Across all health care professions, the primary motivation to enroll was for personal learning to improve clinical practice. Continuing education credit was also an important motivator, particularly for nonphysicians and learners in LICs and LMICs. Course cost (3423/6272, 54.58%) and certification (4238/6272, 67.57%) were also important to a majority of learners.

**Conclusions:**

Our results demonstrate that a diverse range of health care professionals accessed MOOCs for just-in-time training during a public health emergency. Although all health care workers were motivated to improve their clinical practice, different factors were influential across professions and locations. These factors should be considered in MOOC design to meet the needs of health care workers, particularly those in lower-resource settings where alternative avenues for training may be limited.

## Introduction

During the COVID-19 pandemic, massive open online courses (MOOCs) emerged as an invaluable source of training for health care workers globally [1-4]. Studies have demonstrated MOOCs’ effectiveness in facilitating learning among practicing health care professionals [5,6], and their capability to deliver content rapidly and flexibly has established e-learning as a preferred method for transferring clinical skills and knowledge [6]. Their broad applicability, accessibility, and cost-effectiveness make MOOCs particularly appealing for continuing education (CE) requirements, also known as continuing medical education [5,7,8]. Consequently, MOOCs have been used for skill development and retention, competency assessment, and lifelong learning [9]. In low-income countries (LICs) and lower-middle–income countries (LMICs), MOOCs potentially increase access to essential health education content and reduce training costs for health care professionals [5,10,11].

Despite the increasing data on general MOOC enrollee motivations [12-15], there remains a significant gap concerning the specific factors motivating practicing health care professionals. Understanding the motivations of health care workers in LICs and LMICs to enroll in and complete health care–related MOOCs is crucial, as engagement and completion rates among this group are notably low [16-18]. By identifying what drives their participation, we can enhance MOOC design and dissemination, particularly for just-in-time learning initiatives during health emergencies—a time when organizations such as the World Health Organization (WHO) and national governments increasingly rely on MOOCs to rapidly disseminate critical information to health care workers.

This study aims to uncover the characteristics and motivations of health care professionals who enrolled in health care–related MOOCs during the COVID-19 pandemic—a period marked by an urgent need to rapidly disseminate critical health care information. Research indicates several potential reasons for enrolling in MOOCs. As a teaching model, MOOCs support adult learning principles targeting self-directed learners [17]. The self-directed learning model allows individuals to guide their learning process, establish their learning objectives, engage in individualized learning strategies, and manage their time based on their interests while still receiving access to curated content [17]. It can be presumed that learner motivations for engaging in MOOCs differ from those in traditional brick-and-mortar educational venues [19]. Prior studies suggest that primary intrinsic motivations for MOOC enrollment include personal interest and knowledge acquisition [12], whereas extrinsic motivations often involve certification and professional development opportunities [17]. However, the specific motivations driving health care workers, particularly those in LICs and LMICs, remain underexplored.

Although recent studies, such as Garrido et al [20] and a scoping review on MOOCs for health care worker education in low- and middle-income countries [21], have begun to explore the use of MOOCs for professional and workforce development, these insights predominantly focus on broad educational outcomes and employment advancements. Such research underscores the potential of MOOCs to enhance skill sets and career opportunities, highlighting the alignment of MOOC coursework with job market needs and professional certifications. However, these studies generally do not delve deeply into the specific intrinsic motivations of health care workers in LICs and LMICs to enroll in MOOCs, especially during health emergencies. In fact, in 2023, the WHO commissioned 3 systematic reviews of the literature to support guidelines for building just-in-time training during public health emergencies, finding a gap in the literature regarding the motivations of learners enrolling in relevant online courses, particularly in LMICs (WHO, unpublished data, 2023). Our study seeks to fill this void by examining the unique motivations behind MOOC enrollment, particularly during the unprecedented global crisis triggered by the COVID-19 pandemic.

This study contributes uniquely to the literature by investigating the key motivations for health care workers to enroll in MOOCs, with a special emphasis on provider type and country income level during a global health crisis. These insights are vital as learners in LICs and LMICs face challenges such as linguistic and cultural barriers, limited access to digital technology, low-bandwidth connectivity, infrastructure constraints, and limited digital literacy [5,10]. By understanding what motivates learners in these settings, our study provides foundational knowledge that can inform more thoughtful and effective MOOC design and recruitment strategies, ultimately improving knowledge transmission, learning outcomes, and course completion rates in regions with critical needs for health care worker training. This broad impact underscores the potential of targeted online education strategies to significantly enhance global health responses.

## Methods

### Study Design

In this study, we present a descriptive analysis of MOOC learner data to identify the characteristics and motivations of health care workers enrolled in 7 MOOCs designed to serve as just-in-time education for clinically practicing health care workers during the COVID-19 pandemic. We examine two sources of data: (1) course enrollment data (n=49,098) collected during course registration and (2) follow-up survey data (n=6272) collected from course completers.

### Course Descriptions

In Table 1, we detail the 7 focal courses examined in this study. We selected 6 courses developed by the WHO in early 2020 to respond to the growing COVID-19 crisis. These courses were launched on the OpenWHO online platform, which serves as the WHO’s learning hub for health emergencies. These courses build on the WHO’s initial introductory COVID-19 course, which had 232,890 enrollments across 13 published languages by the end of March 2020 and provided general information about the disease for a broad audience [22]. The 6 WHO courses were selected out of all 43 COVID-19 courses offered on the OpenWHO platform due to their greater content relevance to practicing health care workers. The 6 MOOCs focused on introducing health care workers to the novel disease and providing them with strategies to control its outbreak. Three courses were designed to provide health care workers with the basic tools needed to combat the pandemic and protect themselves from infection when providing health care services. Another 3 courses were designed to provide health care workers with an overview of the COVID-19 disease and provide learners with specific clinical strategies to address the pandemic. The courses were initially published in English and then rapidly translated into over 19 languages in the subsequent 2 months.

**Table 1.** Course descriptions.

Source and course title		Description	Languages	Datelaunched	Course duration	Enrolled learners, n
**Stanford University**						
	COVID-19 Training for Healthcare Workers	This course is designed for health care professionals. It provides an evidence-based approach to life-saving techniques for treating critically ill patients with COVID-19.	English, Hindi, Portuguese, French, and Spanish	July 17, 2020	8 h	101,734
**OpenWHO**						
	Hand Hygiene	This course is designed to summarize the WHO^a^ guidelines on hand hygiene, associated tools, and ideas for effective implementation. The WHO guidelines support hand hygiene promotion and improvement in health care facilities worldwide.	Arabic, Chinese, Dutch, English,French, Macedonian, Portuguese, Russian, Shqip, Sinhalese, Somali, Spanish, Tamil, Tetum, and Turkish	June 3, 2020	1 h	274,116
	Personal Protective Equipment	The course is a guide for health care workers involved in patient care activities in a health care setting. It aims to show the type of personal protective equipment needed to correctly protect oneself.	Albanian, Arabic, Chinese, Dutch, English, French, Kazakh, Macedonian, Portuguese, Russian, Sinhalese, Somali, Spanish, Tamil, Tetum, Thai, and Turkish	April 15, 2020	15 min	346,200
	Occupational Health and Safety	This course is for health workers, incident managers, supervisors, and administrators who make policies and protocols for their health facilities. The WHO recommends a combination of measures for infection prevention and control, occupational health and safety, and psychosocial support.	Dutch, English, Indonesian, Macedonian, Portuguese, Spanish, and Swahili	August 30, 2020	1 h	85,504
	Clinical Management: Patient Rehabilitation	The course is devoted to the rehabilitation of patients with COVID-19 by addressing needs of patients recovering from COVID-19, including patients with cognitive impairment, physical deconditioning and weakness, respiratory impairment, swallow impairment, and communication impairment, as well as techniques for rehabilitation.	Chinese, English, French, Macedonian, Russian, and Shqip	January 13, 2021	3 h	22,704
	Clinical Management: General Considerations	This course gives background on the pandemic, discusses facility operations, and addresses COVID-19 pandemic preparedness at all levels of health care provision. It also discusses ethical issues arising during COVID-19 care.	English, Indonesian, Macedonian, and Shqip	October 22, 2020	3 h	31,972
	Clinical Management: Acutely Ill Patients	Designed to prepare and support health providers as they provide emergency care to seriously ill patients with COVID-19, including a systematic approach via the WHO and ICRC^b^ Basic Emergency Care course content.	English, Somali, and Spanish	May 5, 2021	6 h	14,190

a WHO: World Health Organization.

b ICRC: International Committee of the Red Cross.

To broaden the reach of learners in the study, we also included a Stanford University MOOC launched in August 2020 to equip health care workers with timely in-service education, to improve their ability to safely and effectively treat patients with the novel disease [23]. The Stanford MOOC was launched on both the Coursera and edX platforms, 2 US-based MOOC providers founded in 2012 that routinely provide university-level courses on various topics including health. As of November 2020, nearly 900 health-related courses were available on the Coursera platform alone [24]. The Stanford course was first developed in English and then translated into 4 additional languages.

The courses were promoted via their respective institutional networks. No paid advertisements were published. The Stanford course was promoted starting in July 2020, with emails sent to over 100,000 Coursera listserve subscribers. The course was also promoted through a variety of Stanford-affiliated social media channels and online publications, YouTube’s spotlight channel, and direct sharing with a network of health education collaborators throughout the world by Stanford team members. The WHO courses were promoted as each course launched on the WHO website, the OpenWHO platform, and through WHO newsletters and mailing lists.

### Data Collection

**Figure 1.**
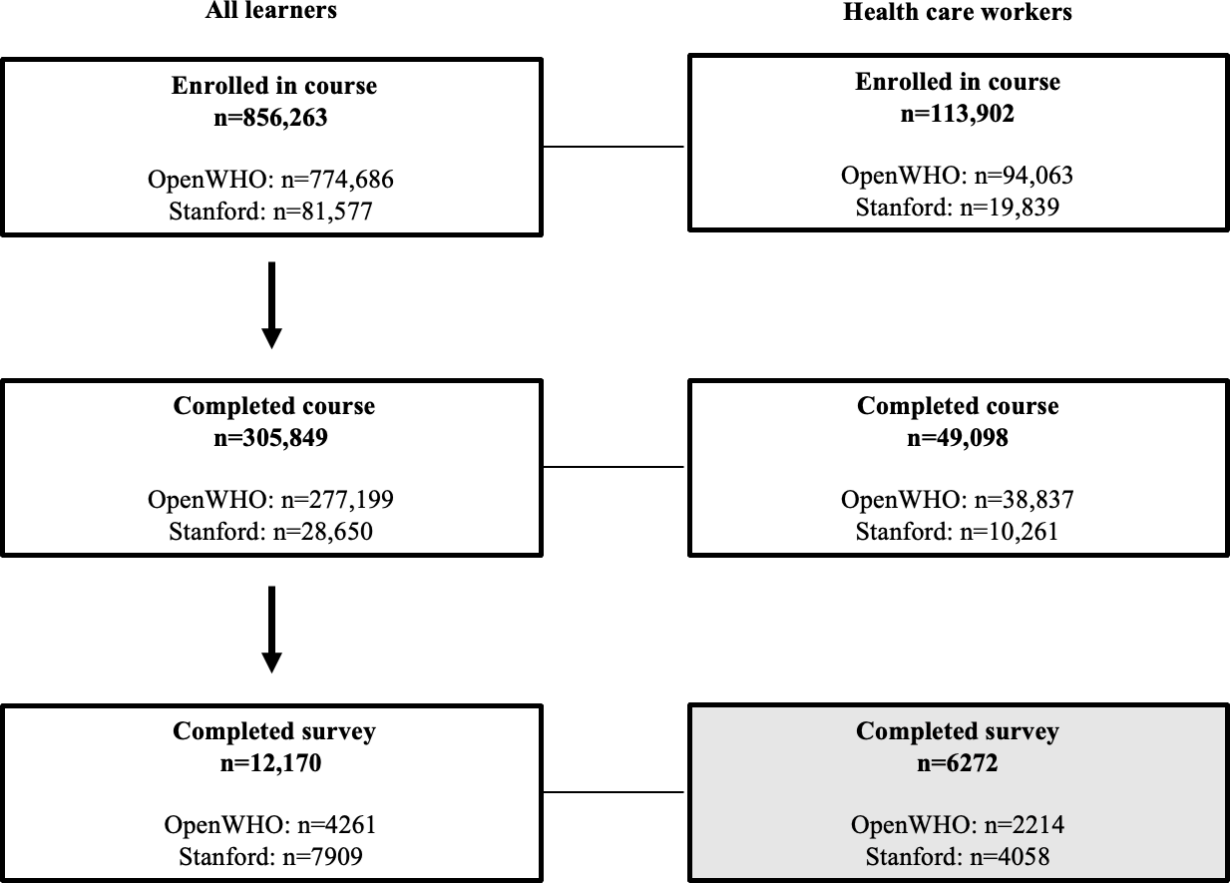
Flow diagram for study participation. The number of enrollees, course completers, and survey completers is shown for all learners and health care workers. The survey completer sample (shaded in gray) is the focal sample for this study. Health care workers included those who identified as being employed as health care professionals at enrollment and in the follow-up survey. Health care professions included the following: allied health; community health; nursing (including nurses, nurse practitioners, nurse midwives, nursing instructors, and certified nursing assistants); physician assistants; paramedics and emergency medical technicians; pharmacy; physicians; and traditional medicine.

Figure 1 describes the flow diagram for study participation and data collection. We obtained data on all course enrollees via the respective course platforms (OpenWHO for WHO courses and edX and Coursera for the Stanford course). Course completion was defined by course developers and identified through backend data available from the course platforms. Learner background data were collected via the respective platforms at the time of course registration and included the learners’ age, gender, geographic location, and profession. The health care worker profession category included those identifying as being employed in the following professions: allopathic medicine (including physicians and physician assistants); traditional medicine; nursing (including nurses, nurse practitioners, nurse midwives, nursing instructors, and certified nursing assistants); allied health (including physical therapy, occupational therapy, speech pathology, medical assistants, and home health aides); community health; emergency medical services (including paramedics and emergency medical technicians); and pharmacy (including pharmacists and pharmacy technicians).

We invited all enrollees who had completed the course they were enrolled in to complete an online survey (Multimedia Appendix 1) on the respective course platforms. To recruit WHO course learners, we sent 3 survey invitations to the email addresses provided by learners at the time of registration and through the OpenWHO automated course message. To recruit participants from the Stanford course, we sent 3 requests via Coursera and edX email announcements. The survey window was open from December 11, 2020, to September 28, 2021. The survey completion rate was 3.98% (12,170/305,849) among all course completers and 12.77% (6272/49,098) among health care workers completing the courses.

The 23-question survey collected information on learners’ personal and professional demographics, information about their professional experience with COVID-19, and their ability to connect with physicians in their daily work. Respondents were asked to rank 6 possible motivations for course enrollment in the order of importance to them. Additionally, respondents were asked about their use of course certificates, including whether their employer required a certificate, if they planned to provide it to their employer, or if they planned to use it for CE credit. Finally, respondents were asked about the cost of MOOCs and how it impacted their decision to enroll in the course. All study authors were involved in the development of the survey. Questions were reviewed by all authors to include appropriate vocabulary, inclusive of globally used terminology. The survey was not adapted directly from any other source; however, the motivations included were drawn from anecdotal course feedback and the extant literature discussing motivations for MOOC enrollment.

### Statistical Analysis

Because of the study focus, we limited our analytic sample to health care workers exclusively. To investigate the generalizability of our survey sample, we summarized the characteristics of all health care workers completing the courses (n=49,098) and health care workers completing the survey (n=6272) using descriptive statistics (mean, SD, and response rates). To compare the proportion of learners by characteristic between course completers and survey completers, we used the Pearson *χ*^2^ test. To examine ranked enrollment motivators and compare across learner subgroups, we conducted multiple comparison tests using 1-way ANOVA, comparing the mean rank of motivations (dependent variable) by learner characteristics. The independent variables compared included differences by occupation (physicians vs nurses and physicians vs other health professionals) and country income classification (LICs and LMICs vs upper-middle–income countries [UMICs] and high-income countries [HICs]). All statistical analyses were conducted using Stata SE V15 (StataCorp).

### Ethical Considerations

Informed consent was obtained from all learners. Participation was voluntary and no monetary compensation was provided to the participants. The collected data were anonymized. Approval for all aspects of this study design, including consent, outreach, data collection, surveying, and data analysis, was obtained from the Stanford University School of Medicine Institutional Review Board (protocol 57831).

## Results

### Learner Characteristics

As shown in Figure 1, as of September 2021, the 7 courses had 856,263 total enrollees, 90.47% (n=774,686) in WHO courses and 9.53% (n=81,577) in the Stanford course. In all, 13.3% (113,902/856,263) of enrollees and 16.05% (49,098/305,849) of course completers identified as practicing health care workers at course registration. The course completion rate was higher among health care workers (49,098/113,902, 43.1%) than overall enrollees (305,849/856,263, 35.72%).

Table 2 shows that nearly one-third (15,238/49,098, 31.04%) of the health care workers that completed a course were between the ages of 18‐29 years, and 41.25% (20,252/49,098) identified as female. The region with the most health care workers that completed a course was Latin America and the Caribbean (10,665/49,098, 21.72%), followed by South Asia (7264/49,098, 14.79%), North America (7019/49,098, 14.3%), Europe and Central Asia (5365/49,098, 10.93%), East Asia and the Pacific (5278/49,098, 10.75%), Middle East and North Africa (3816/49,098, 7.77%), and sub-Saharan Africa (3502/49,098, 7.13%). Nearly one-third (14,159/49,098, 28.84%) of the health care workers who completed a course were from LICs (828/49,098, 1.69%) or LMICs (13,331/49,098, 27.15%).

**Table 2.** Health care worker characteristics, by course and survey completion. This table compares the characteristics of health care workers who completed the focal courses and follow-up survey. A higher proportion of course completers did not specify characteristics compared to survey completers. Because response options for age and gender were voluntary, a number of learners did not specify these characteristics. We show the numbers not specified for each. For course completion, geographic region was identified via course platform analytics; however, we were unable to identify a subset, shown as "not specified" in the table. For survey completion, geographic regions were identified primarily through survey self-reports. In 177 survey responses, location was not reported. For these cases, we used the survey response’s IP address to identify the geographic region of the respondent. Percentages are shown for those for whom we have data on characteristics. Percentage for each categorical variable sum to 100.

Characteristics		Completed course (n=49,098), n (%)	Completed survey (n=6272), n (%)	*P* value
**Course type**				
	OpenWHO	38,837 (79.1)	2214 (35.3)	<.001
	Stanford University	10,261 (20.9)	4058 (64.7)	<.001
**Age range (y)**				
	18‐29	15,238 (31.04)	2020 (32.21)	<.001
	30‐39	9699 (19.75)	1560 (24.87)	.10
	40‐49	4511 (9.19)	950 (15.15)	<.001
	50‐59	2324 (4.73)	662 (10.55)	<.001
	60‐69	691 (1.41)	232 (3.7)	<.001
	70+	233 (0.47)	35 (0.56)	.56
	Not specified	16,402 (33.41)	813 (12.96)	—^a^
**Gender**				
	Female	20,252 (41.25)	3057 (48.74)	<.001
	Male	12,758 (25.98)	2349 (37.45)	<.001
	Nonbinary or other	139 (2.83)	43 (0.69)	<.001
	Not specified	15,949 (32.48)	823 (13.12)	—
**Geographic region**				
	East Asia and Pacific	5278 (10.75)	894 (14.25)	<.001
	Europe and Central Asia	5365 (10.93)	666 (10.62)	<.001
	Latin America and Caribbean	10,665 (21.72)	1061 (16.92)	<.001
	Middle East and North Africa	3816 (7.78)	547 (8.72)	.66
	North America	7019 (14.3)	993 (15.83)	.29
	South Asia	7264 (14.79)	1393 (22.21)	<.001
	Sub-Saharan Africa	3502 (7.13)	718 (11.45)	<.001
	Not specified	6189 (12.61)	0 (0)	—
**World Bank income classification**				
	High income	14,157 (28.83)	1971 (31.43)	.01
	Upper-middle income	14,593 (29.72)	1611 (25.69)	<.001
	Lower-middle income	13,331 (27.15)	2468 (39.35)	<.001
	Low income	828 (1.69)	222 (3.54)	<.001
	Not specified	6189 (12.61)	0 (0)	—

a Not applicable.

Table 2 also compares the characteristics of health care workers completing the course, with the 12.77% (6272/49,098) completing the survey. We observe slight differences in the age and gender composition of survey completers with course completers, with the survey sample skewing older and more male. The survey sample includes a slightly larger share of participants from LICs (222/6272, 3.54%) and LMICs (2468/6272, 39.35%).

Table 3 describes the professions of the health care workers who completed the survey and their levels of physician supervision. Physicians represent 34.61% (2171/6272) of the survey sample, followed by nurses (1599/6272, 25.49%) and allied health professionals (1190/6272, 18.97%). This breakdown of professional roles is similar in LICs and LMICs and in UMICs and HICs. Of the nonphysician health care workers, more than a third (1315/3639, 36.14%) reported having access to a physician for consultation during less than 50% of their workday, although the majority (1989/2341, 84.96%) could contact a physician by phone if needed. Most health care workers either already cared for patients with COVID-19 (2793/6272, 44.53%) or anticipated caring for them (1940/6272, 30.93%) at the time of survey completion.

**Table 3.** Characteristics of the health care worker survey sample. Allied health included physical therapy, occupational therapy, speech pathology, medical assistants, and home health aides. Nursing included nurses, nurse midwives, nursing instructors, and certified nursing assistants. The question about the frequency of physicians being on site was asked of nonphysicians only. The question about physicians being available via phone was asked of nonphysicians who had indicated that physicians were not available on site 100% of the time. Across questions asking about the availability of physician and treating patients with COVID-19, survey respondents could indicate that the question was not applicable in their health care setting.

Characteristics		Total (n=6272), n (%)	HICs^a^ and UMICs^b^ (n=3582), n (%)	LMICs^c^ and LICs^d^ (n=2690), n (%)
**Profession**				
	Allied health	1190 (18.97)	663 (18.51)	527 (19.59)
	Community health worker	501 (7.99)	296 (8.26)	205 (7.62)
	Nursing	1599 (25.49)	1012 (28.25)	587 (21.82)
	Physician assistant or nurse practitioner	103 (1.64)	68 (1.9)	35 (1.3)
	Paramedic or emergency medical technician	272 (4.34)	159 (4.44)	113 (4.2)
	Pharmacist	330 (5.26)	106 (2.96)	224 (8.33)
	Physician	2171 (34.61)	1217 (33.98)	954 (35.46)
	Traditional medicine	106 (1.69)	61 (1.7)	45 (1.67)
**Frequency of physicians being on site** ^e^				
	Always (100% of time)	1228 (33.75)	660 (30.88)	568 (37.82)
	Mostly (>50% of time)	1096 (30.12)	586 (27.42)	510 (33.95)
	Sometimes (<50% of time)	815 (22.4)	482 (22.55)	333 (22.17)
	Never (0% of time)	500 (13.74)	409 (19.14)	91 (6.06)
**Physicians being available via phone** ^f^				
	Yes	1989 (82.5)	1180 (48.94)	809 (33.55)
	No	352 (14.6)	256 (10.62)	96 (3.98)
	Not specified	70 (2.9)	41 (1.7)	29 (1.2)
**Treating patients with COVID-19** ^g^				
	Currently treating	2793 (44.53)	1551 (43.3)	1242 (46.17)
	Anticipated in future	1940 (30.93)	1003 (28)	937 (34.83)
	Not anticipated	460 (7.33)	314 (8.77)	146 (5.43)
	Not specified	1079 (17.2)	714 (19.93)	365 (13.57)

a HIC: high-income country.

b UMIC: upper-middle–income country.

c LMIC: lower-middle–income country.

d LIC: low-income country.

e This survey question was only asked to nonphysician health care workers who work directly with physicians (n=3639). Percentages shown are out of applicable participants only.

f This survey question was only asked to nonphysician health care workers that work directly with physicians and do not have a physician on site 100% of the time (n=2411). Percentages shown are out of data provided with applicable respondents only. Not all applicable respondents responded to this question (n=70).

g Data on whether health care workers treat patients with COVID-19 were based on a voluntary question asked of patients at the time of course enrollment.

### Learner Motivations

**Figure 2.**
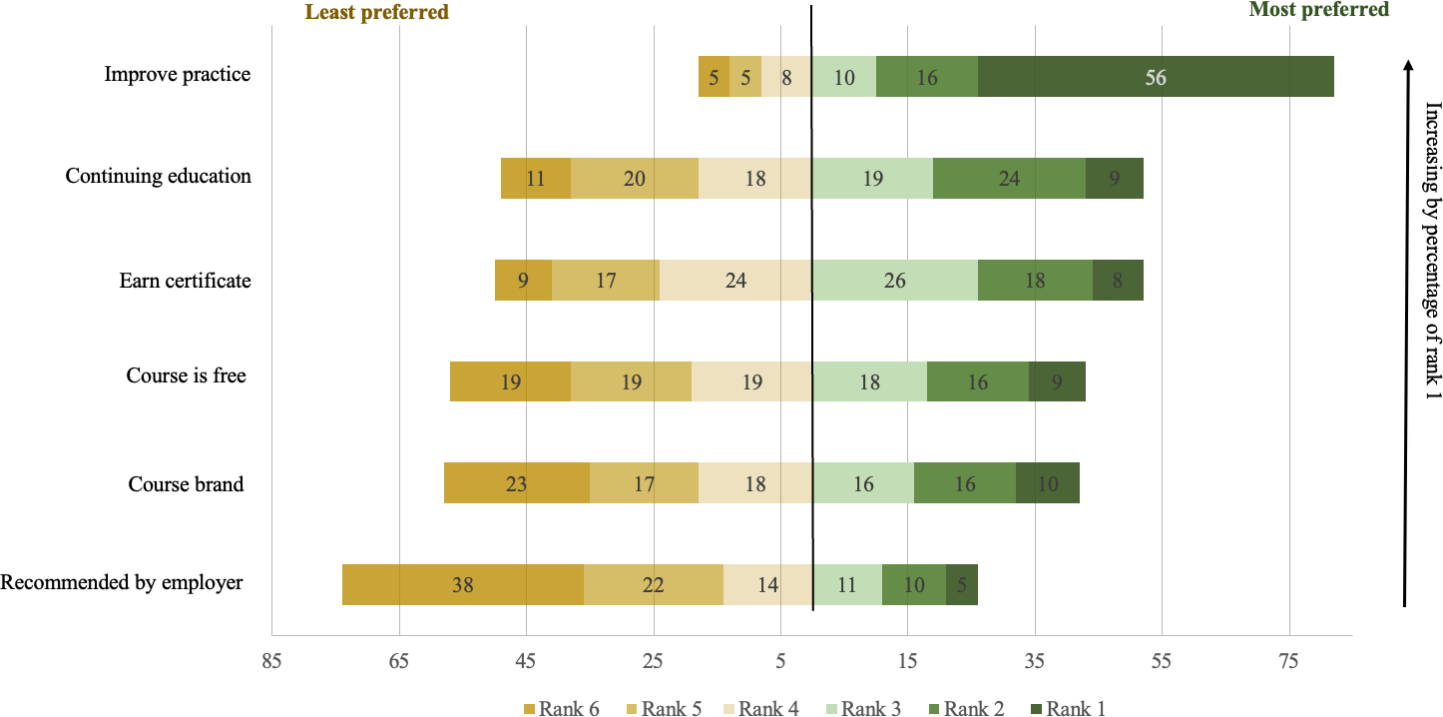
Percent of learners by motivation rank among health care providers (n=5518).

In the survey, health care workers were asked to rank in importance the following 6 potential motivating factors for course enrollment: to improve practice, to earn a certificate, CE, course brand, free cost of course, and employer recommendation. Figure 2 shows the ranking preferences across survey respondents. Among survey respondents ranking all factors (n=5518), the majority (n=3090, 56%) ranked “improve practice” as their top preference, with an additional 16% (n=883) ranking it as the second most important factor and 10% (n=552) ranking it as the third most important factor. The second and third most important factors were CE and to earn a certificate, with employer recommendation as the least most important factor ranked.

In Table 4, we show the ranking differences by the type of health care worker. Although the motivation of improving practice was ranked the highest across all subgroups, it was ranked higher by physicians, with a mean rank of 1.86, compared to nurses with a mean rank of 2.06 and other health care providers with a mean rank of 2.24. Nonphysicians ranked CE and employer recommendations higher than physicians. Certification also appears to matter more to nonphysicians, with 69.76% (2861/4101) choosing to obtain a certificate, 63.76% (2615/4101) providing a copy of the certificate to their employer, and 79.18% (3247/4101) using the certificate for a CE requirement. The course brand appears to be a more important motivating factor to physicians compared to nonphysicians. Course cost did not appear to differentially influence course enrollment by the type of health care worker.

**Table 4.** Mean rank of motivation (1=highest rank, 6=lowest rank) and course perspectives by the type of health care worker. Physician is the reference category for comparisons. Nursing included nurses, nurses, midwives, and nursing assistants. Mean ranking does not include observations that skipped ranking altogether (n=745). Course perspectives include observations that skipped ranking but provided responses for these questions.

		Physician (n=2171), mean (SD)	Nursing (n=1599), mean (SD)	*P* value	Other (n=2502), mean (SD)	*P* value
**Motivation (mean ranking)**						
	Improve practice	1.86 (1.38)	2.06 (1.51)	<.001	2.24 (1.60)	<.001
	Earn certificate	3.52 (1.36)	3.53 (1.36)	.80	3.46 (1.42)	.16
	Continuing education requirement	3.63 (1.49)	3.31 (1.47)	<.001	3.46 (1.55)	<.001
	Course brand	3.58 (1.61)	4.17 (1.62)	<.001	3.92 (1.68)	<.001
	Course is free	3.83 (1.57)	3.77 (1.65)	.27	3.81 (1.61)	.68
	Employer recommended	4.66 (1.54)	4.47 (1.55)	.001	4.39 (1.61)	<.001
**Course perspectives (proportion agreeing)**						
	Would have taken course if it was not free	0.47 (0.50)	0.43 (0.50)	.01	0.46 (0.50)	.39
	Chose to obtain a certificate	0.63 (0.48)	0.71 (0.46)	<.001	0.69 (0.46)	<.001
	Gave a copy of the certificate to employer	0.55 (0.50)	0.65 (0.48)	<.001	0.63 (0.48)	<.001
	Will use the certificate for continuing education requirement	0.71 (0.45)	0.81 (0.39)	<.001	0.78 (0.41)	<.001

In Table 5, we show ranking differences by the location of health care workers, comparing differences in UMICs and HICs compared to LICs and LMICs. In LICs and LMICs, health care workers ranked CE and employer recommendation higher on average compared to learners in UMICs and HICs. Conversely, course brand appears to matter more for learners in UMICs and HICs. Certification was obtained by roughly the same proportion of learners in both subgroups, although learners in UMICs and HICs were more likely to give a copy of the certificate to their employer, whereas learners in LICs and LMICs were more likely to use the certificate for a CE requirement.

**Table 5.** Mean rank of motivation (1=highest rank, 6=lowest rank) and course perspectives by country classification. This table shows differences by World Bank income classifications: high-income country (HIC), upper-middle–income country (UMIC), lower-middle–income country (LMIC), and low-income country (LIC). Mean ranking does not include observations that skipped ranking altogether (n=745). Course perspectives include observations that skipped ranking but provided responses for these questions.

		HICs and UMICs (n=3582), mean (SD)	LICs and LMICs (n=2690), mean (SD)	*P* value
**Motivation (mean ranking)**				
	Improve practice	2.10 (1.52)	2.01 (1.49)	.04
	Earn certificate	3.45 (1.38)	3.57 (1.38)	.001
	Continuing education requirement	3.58 (1.54)	3.37 (1.48)	<.001
	Course brand	3.77 (1.65)	3.97 (1.66)	<.001
	Course is free	3.68 (1.59)	3.97 (1.61)	<.001
	Employer recommended	4.58 (1.58)	4.41 (1.57)	<.001
**Course perspectives (proportion agreeing)**				
	Would have taken course if it was not free	0.45 (0.50)	0.46 (0.50)	.61
	Chose to obtain a certificate	0.68 (0.47)	0.67 (0.47)	.22
	Gave a copy of the certificate to employer	0.65 (0.48)	0.57 (0.50)	<.001
	Will use the certificate for continuing education requirement	0.73 (0.44)	0.81 (0.39)	<.001

Generally, the fact that MOOCs were free was a lower-ranked motivator. Although interestingly, in the subgroup analysis, the course being free of cost was ranked lower in LICs and LMICs (mean 3.97, SD 1.61) than in UMICs and HICs (mean 3.68, SD 1.59; Table 5). However, when survey respondents were asked about their perspectives on the cost of MOOCs, more than half (3423/6272, 54.58%) of the health care workers indicated they would not have taken the course if there was an associated cost. This perspective was consistent across subgroup analyses of health care professional types and country-income levels.

## Discussion

### Principal Findings

Through a survey of 6272 health care workers worldwide who completed MOOCs for COVID-19 training across multiple platforms and organizations, our study provides unique insight into the factors motivating health care workers to enroll in and complete MOOCs during public health emergencies. We identified that the primary motivator for enrollment among health care workers was to improve their personal practice, followed by the pursuit of CE credit and certification. Course cost is an influential factor in the decision to enroll in an MOOC, with 54.58% (3423/6272) of respondents indicating that they would not have enrolled if the course had not been free. This first-of-its-kind analysis of health care worker motivations in just-in-time training MOOCs during a public health emergency fills an important gap in the existing literature, providing key insights for future course development and marketing.

Our findings highlight the widespread demand among health care workers for MOOC training during a public health crisis. Health care workers from over 200 countries and territories enrolled in and completed the COVID-19 MOOCs examined in this study, with a third (14,159/42,909, 33%) of course completers located in LICs and LMICs. Compared to the typical MOOC completion rates of under 10% [17,18], the 43.1% (49,098/113,902) completion rate among health care workers in the COVID-19 MOOCs in this study is notably high. Although the high rate of completion likely reflects the limited alternatives for training during the start of the COVID-19 pandemic, it may also indicate intrinsic motivation among health care workers, whose predominant reason for enrollment was to improve their personal practice.

We also observed that the COVID-19 MOOCs attracted a diverse range of health care providers globally. Although the majority (3770/6272, 60.11%) of respondents were nurses and physicians, 39.89% (2502/6272) reported working in other health care capacities including allied health, community health, emergency medical services, and pharmacy. Furthermore, we noted that motivations for enrollment varied by profession. Compared to physicians, nurses and other health care professionals were more motivated by CE credit, employer recommendations, and certification. Nurses and other health professionals were more likely to obtain certificates, provide a copy of the certificate to their employer, and use the certificates for CE requirements. Recognizing these differences in motivating factors across types of health care workers can inform the design of MOOCs that more effectively respond to the interests and needs of the targeted audience.

Despite these differences, the majority of all health care workers, including physicians, indicated their intention to use their certificates professionally, either by providing them to their employers (3809/6272, 60.73%) or by earning CE credit (4788/6272, 76.34%). This finding underscores the potential for MOOCs to fill a gap in the CE arena, where traditional approaches often present barriers to completion. The common, traditional route for obtaining CE credits involves attendance at national or international medical conferences [7,8]; however, many such conferences were either canceled or transitioned to a web-based format during the pandemic. Given the time and travel requirements associated with conference attendance, MOOCs can serve as a viable and accessible alternative for learners. Interestingly, our study found that the use of course certificates for CE among learners in LICs and LMICs was higher than that in UMICs and HICs, which may reflect a lack of economically feasible options to earn CE credits in resource-limited geographies. Including certification in MOOC design may serve as an important motivator to increase enrollment and completion, particularly in LICs and LMICs, enhancing the attainment of timely health care education for the global health care workforce.

An additional benefit of online learning is the reduced cost for participants to obtain CE credits. Our study found that cost was a significant consideration for course participants, with 54.58% (3423/6272) of learners indicating they would not have taken the course if it had not been free. Although the course being free was slightly less important to learners in LICs and LMICs than those in UMICs and HICs, we speculate that in lower-income countries, learners with access to the technology required to participate in an online course may be relatively better off financially within their respective countries, and that those with lower incomes may not have the technology to enroll in the courses at all—only 3.54% (222/6272) of learners were from LICs. It is also possible that a single course participant may have shared access to the course with others.

Identifying the characteristics and motivations of specific groups of learners, such as those in LICs and LMICs, will aid in the design of future health care–related MOOCs to encourage participation and completion. Although many public health emergencies and disease outbreaks occur in LICs and LMICs with devastating impact, little data exist that examine the motivations of health care workers in these regions to enroll in just-in-time training MOOCs. Nevertheless, the WHO and various national health agencies frequently leverage MOOCs to disseminate critical health information during these emergencies. Future work should particularly investigate how to overcome barriers related to technology access and content accessibility with an eye toward equity, ensuring that the delivery of crucial health care worker training, particularly in times of emergency, is available to all. Likewise, future investigations should examine how online content is used and shared offline in contexts where the broader population has limited access to digital platforms, thereby enhancing the delivery of course materials through offline sharing.

### Limitations

We recognize several methodological limitations inherent in our survey-based research. First, the potential for social desirability bias and selection bias due to voluntary participation limits the generalizability of our findings. To mitigate these biases, we deployed the survey across multiple learning platforms (Coursera, edX, and OpenWHO), each likely attracting different user demographics, and achieved a substantial sample size of 6272 respondents representing a diverse economic and geographic distribution. Additionally, we examined and reported only marginal differences between survey respondents and the overall course participants (as detailed in Table 2), although it remains a limitation that survey completers may not fully represent the broader learner population.

Second, the exclusive use of English for survey dissemination likely influenced the diversity of the respondents and further constrained the study’s generalizability. Future studies could incorporate multiple language options to better capture a wider demographic.

Third, although the survey instrument was tailored to the specific contexts of the courses and discussed rigorously by experts across various fields—including educational assessment, emergency medicine, public health, and online learning—its lack of external validation presents a limitation. No prior studies identified during our review provided a validated instrument for assessing learner motivations in MOOCs, emphasizing the innovative aspect of our research while also necessitating a careful interpretation of our findings.

Fourth, our study’s scope was restricted by the limitations in identifying patient-facing health care workers among enrollees, due to data collection methodologies on the OpenWHO platform until June 2020. This limitation hindered our capability to fully classify professions among participants. Future studies should aim to enhance the categorization of health care worker types and delve deeper into the differing motivations among these groups.

Finally, the dynamics of the COVID-19 pandemic—characterized by fluctuating case rates and mortality—suggest that motivations for enrolling in COVID-19–related MOOCs likely varied over time. Some health care workers might have enrolled early in anticipation of patient care needs, whereas others joined after gaining firsthand experience. This temporal variation in motivations, coupled with the evolving availability of other educational tools, presents a complex backdrop against which these motivations were formed. Future studies could benefit from aligning course enrollment data with local COVID-19 case trends to better understand these motivations.

### Conclusion

Our study examined the motivations and characteristics of health care workers who engaged with MOOCs during the unprecedented COVID-19 health emergency. The analysis showed that the primary motivation for health care professionals was enhancing their personal practice. CE credit also proved to be a significant motivator, especially for those from LICs and LMICs. Additionally, the necessity of free access was clear, with more than half of the participants (3423/6272, 54.58%) indicating they would not have enrolled if fees were charged. These findings are important for the future development and deployment of MOOCs, ensuring that they not only are accessible but also resonate with the intrinsic and extrinsic motivations of health care professionals from diverse geographic, training, and economic backgrounds. Future research should further investigate these motivations to see if they are consistent across different types and stages of health emergencies.

## Supplementary material

10.2196/51915 Multimedia Appendix 1 COVID-19 provider course: follow-up survey.

## Abbreviations

CE: continuing education
HIC: high-income country
LIC: low-income country
LMIC: lower-middle–income country
MOOC: massive open-source online course
UMIC: upper-middle–income country
WHO: World Health Organization

## References

[1] Utunen H, George R, Ndiaye N, Attias M, Piroux C, Gamhewage G (2020). Responding to global learning needs during a pandemic: an analysis of the trends in platform use and incidence of COVID-19. Educ Sci.

[2] Findyartini A, Greviana N, Hanum C (2021). Supporting newly graduated medical doctors in managing COVID-19: an evaluation of a massive open online course in a limited-resource setting. PLoS One.

[3] Bendezu-Quispe G, Torres-Roman JS, Salinas-Ochoa B, Hernández-Vásquez A (2017). Utility of massive open online courses (MOOCs) concerning outbreaks of emerging and reemerging diseases. F1000Res.

[4] Bhattacharya S, Singh A, Hossain MM (2020). Health system strengthening through massive open online courses (MOOCs) during the COVID-19 pandemic: an analysis from the available evidence. J Educ Health Promot.

[5] Liyanagunawardena TR, Aboshady OA (2018). Massive open online courses: a resource for health education in developing countries. Glob Health Promot.

[6] Regmi K, Jones L (2020). A systematic review of the factors - enablers and barriers - affecting e-learning in health sciences education. BMC Med Educ.

[7] Setia S, Tay JC, Chia YC, Subramaniam K (2019). Massive open online courses (MOOCs) for continuing medical education - why and how?. Adv Med Educ Pract.

[8] Furtner D, Shinde SP, Singh M, Wong CH, Setia S (2022). Digital transformation in medical affairs sparked by the pandemic: insights and learnings from COVID-19 era and beyond. Pharm Med.

[9] Mahajan R, Gupta P, Singh T (2019). Massive open online courses: concept and implications. Indian Pediatr.

[10] King M, Pegrum M, Forsey M (2018). MOOCs and OER in the Global South: problems and potential. The International Review of Research in Open and Distributed Learning.

[11] Perryman LA, Hemmings-Buckler A, Seal T (2014). Learning from TESS-India’s approach to OER localisation across multiple Indian States. J Interact Media Educ.

[12] Bayeck RY (2016). Exploratory study of MOOC learners’ demographics and motivation: the case of students involved in groups. Open Praxis.

[13] Christensen G, Steinmetz A, Alcorn B, Bennett A, Woods D, Emanuel EJ (2014). The MOOC phenomenon: who takes massive open online courses and why?. SSRN.

[14] Zhong SH, Zhang QB, Li ZP (2016). Motivations and challenges in MOOCs with Eastern insights. Int J Inf Educ Technol.

[15] Hew KF, Cheung WS (2014). Students’ and instructors’ use of massive open online courses (MOOCs): motivations and challenges. Educ Res Rev.

[16] Kizilcec RF, Piech C, Schneider E, Suthers D, Verbert K, Duval E (2013). LAK ’13: Proceedings of the Third International Conference on Learning Analytics and Knowledge.

[17] Maya-Jariego I, Holgado D, González-Tinoco E, Castaño-Muñoz J, Punie Y (2020). Typology of motivation and learning intentions of users in MOOCs: the MOOCKNOWLEDGE study. Educ Technol Res Dev.

[18] Reich J, Ruipérez-Valiente JA (2019). The MOOC pivot. Science.

[19] Kizilcec RF, Schneider E (2015). Motivation as a lens to understand online learners: towards data-driven design with the OLEI scale. ACM Transac Comput Hum Int.

[20] Garrido M, Koepke L, Anderson S, Mena A, Macapagal M, Dalvit L (2016). An examination of MOOC usage for professional workforce development outcomes in Colombia, the Philippines, & South Africa. Technology & Social Change Group, University of Washington Information School.

[21] Nieder J, Nayna Schwerdtle P, Sauerborn R, Barteit S (2022). Massive open online courses for health worker education in low- and middle-income countries: a scoping review. Front Public Health.

[22] Utunen H, Ndiaye N, Piroux C, George R, Attias M, Gamhewage G (2020). Global reach of an online COVID-19 course in multiple languages on OpenWho in the first quarter of 2020: analysis of platform use data. J Med Internet Res.

[23] COVID-19: training for healthcare workers. Stanford Online.

[24] Top healthcare courses - learn healthcare online. Coursera.

