# Supplementary material for: Health Care Workers’ Motivations for Enrolling in Massive Open Online Courses During a Public Health Emergency: Descriptive Analysis
Source: JMIR Med Educ. 2024 Jun 19;10:e51915. doi: 10.2196/51915 (PMC11199926; doi:10.2196/51915)
Supplement: Multimedia Appendix 1 [file mededu-v10-e51915-s001.docx]

**COVID-19 PROVIDER COURSE: FOLLOW-UP SURVEY**

Objective: To determine the importance of earning the course certificate in participants’ motivation to take this COVID-19 online educational course.

1. What is your current profession? (Select all that apply)
   1. Community health worker
   2. Health-related researcher
   3. Nurse
   4. Nurse midwife
   5. Nursing assistant
   6. Paramedic or EMT
   7. Pharmacist
   8. Physician
   9. Physician assistant or nurse practitioner
   10. Public health practitioner
   11. Student – specify degree path:
       1. Community health worker
       2. Health-related researcher
       3. Nurse
       4. Nurse midwife
       5. Nursing assistant
       6. Paramedic or EMT
       7. Pharmacist
       8. Physician
       9. Physician assistant or nurse practitioner
       10. Public health practitioner
       11. Traditional or complementary medicine practitioner
       12. Other healthcare (please specify): [free text]
       13. Other non-healthcare (please specify): [free text]
   12. Traditional or complementary medicine practitioner
   13. Other healthcare (please specify): [free text]
   14. Other non-healthcare (please specify): [free text]
2. In what type of setting do you usually work?
   1. Home care
   2. Hospital
   3. Long term care facility or nursing home
   4. Outpatient clinic or pharmacy
   5. Telehealth
   6. Other (please specify): [free text]
   7. N/A
3. In what part of the hospital do you usually work? (Select all that apply)

(if answer 2 to question 2)

- 1. Emergency department
  2. Intensive care unit
  3. Operating theater
  4. Wards
  5. Other (please specify): [free text]

1. In your usual setting, how often do you have a physician available for in-person guidance? (only for non-physician, non-student healthcare workers question 1)
   1. Always (100%)
   2. Mostly (>50%)
   3. Occasionally (<50%)
   4. Never (0%)
2. When a physician is not on site, is one available by telephone?

(only for answer 2-4 question 4)

- 1. Yes
  2. No

1. Do you currently work with COVID-19 patients?
   1. Yes
   2. No
2. Do you anticipate that you will work with COVID-19 patients in the future?
   1. Yes
   2. No
3. Please rank in order of importance the following reasons you considered when deciding to take this course? (1 being most important, 6 being least important) [Rank order]
   1. Course is free (Coursera)
   2. Earn the certificate for course completion
   3. Fulfill continuing education requirements
   4. Improve clinical practice/personal learning
   5. Recommended by my employer/institution
   6. Stanford University designed course
4. Would you have taken this course if it had not been free? (Coursera learners)
   1. Yes
   2. No
5. Would you have taken this course if it had cost more? (EdX learners)
   1. Yes
   2. No
6. Did you choose to earn the course certificate?
   1. Yes
   2. No
7. Did you give a copy of the course certificate to your employer/institution?

(if answer 1 to question 11)

- 1. Yes
  2. No
  3. Not sure

1. Did your employer/institution reimburse the cost of the course certificate? (EdX learners only)
   1. Full reimbursement
   2. Partial reimbursement
   3. No reimbursement
2. Will you use the certificate to fulfill continuing education requirements?
   1. Yes
   2. No
   3. Not sure
3. How strongly do you agree or disagree with the following statements?
   1. This course has helped change how I will approach my practice
   2. I have received adequate training on managing patients with COVID-19
   3. I feel confident I can recognize clinical signs of COVID-19
   4. I feel confident I can assess patients with difficulty breathing.
   5. I feel confident I can care for COVID-19 patients who may need supplemental oxygen or therapeutics

Options (list separately for each of the above):

Strongly disagree

Somewhat disagree

Somewhat agree

Strongly agree

Prefer not to answer

1. What country do you usually practice in?
   1. (Drop down menu)
2. What zip code do you practice in? (if answer US to 16)
   1. [Numeric]
3. How old are you?
   1. 18-29 years
   2. 30-39 years
   3. 40-49 years
   4. 50-59 years
   5. 60-69 years
   6. 70 years or older
   7. Prefer not to answer
4. What best describes your gender?
   1. Male
   2. Female
   3. Non-binary/third gender
   4. Prefer to self-describe
   5. Other [free text]
   6. Prefer not to answer
5. What is your race or ethnicity?  Select all that apply.
   1. Arab
   2. Black, African, or African American
   3. East Asian
   4. Hispanic, Latinx or Spanish Origin
   5. Native American, Native Alaskan or Indigenous
   6. Native Hawaiian or Pacific Islander
   7. South Asian
   8. Southeast Asian
   9. White or Caucasian
   10. Other [free text]
   11. Prefer not to answer
6. How did you hear about the COVID-19 course?
   1. Colleague
   2. Employer
   3. Friend
   4. Coursera emails and marketing
   5. EdX emails and marketing
   6. Stanford emails and marketing
   7. Internet - please specify site [free text]
   8. Other [free text]
7. Did you recommend this course to others?
   1. Yes
   2. No
8. Please provide any additional comments you have regarding the value of earning the certificate for this course.

[Optional Free text]
